# Public Views on Medicaid Work Requirements and Mandatory Premiums in Kentucky

**DOI:** 10.1001/jamahealthforum.2023.3656

**Published:** 2023-10-20

**Authors:** Kristen Underhill, Elizabeth F. Bair, Erica L. Dixon, William J. Ferrell, Kristin A. Linn, Kevin G. Volpp, Atheendar S. Venkataramani

**Affiliations:** 1 Cornell Law School, Ithaca, New York; 2 Department of Population Health Sciences, Weill Cornell Medical College, New York, New York; 3 Center for Health Incentives and Behavioral Economics, University of Pennsylvania, Philadelphia; 4 Department of Medical Ethics & Health Policy, Perelman School of Medicine, University of Pennsylvania. Philadelphia; 5 Wharton School, University of Pennsylvania, Philadelphia; 6 Corporal Michael J. Cresencz Department of Veterans Affairs Medical Center, Philadelphia, Pennsylvania; 7 Department of Medicine, Perelman School of Medicine, University of Pennsylvania, Philadelphia

## Abstract

**Importance:**

Federal and state policymakers continue to pursue work requirements and premiums as conditions of Medicaid participation. Opinion polling should distinguish between general policy preferences and specific views on quotas, penalties, and other elements.

**Objective:**

To identify views of adults in Kentucky regarding the design of Medicaid work requirements and premiums.

**Design, Setting, and Participant:**

A cross-sectional survey was conducted via telephone and the internet from June 27 through July 11, 2019, of 1203 Kentucky residents 9 months before the state intended to implement Medicaid work requirements and mandatory premiums. Statistical analysis was performed from October 2019 to August 2023.

**Main Outcomes and Measures:**

Agreement, disagreement, or neutral views on policy components were the main outcomes. Recruitment for the survey used statewide random-digit dialing and an internet panel to recruit residents aged 18 years or older. Findings were weighted to reflect state demographics. Of 39 110 landlines called, 209 reached an eligible person (of whom 150 participated), 8654 were of unknown eligibility, and 30 247 were ineligible. Of 55 305 cell phone lines called, 617 reached an eligible person (of whom 451 participated), 29 951 were of unknown eligibility, and 24 737 were ineligible. Internet recruitment (602 participants) used a panel of adult Kentucky residents maintained by an external data collector.

**Results:**

Percentages were weighted to resemble the adult population of Kentucky residents. Of the participants in the study, 52% (95% CI, 48%-55%) were women, 80% (95% CI, 77%-82%) were younger than 65 years, 41% (95% CI, 38%-45%) were enrolled in Medicaid, 36% (95% CI, 32%-39%) were Republican voters, 32% (95% CI, 29%-36%) were Democratic voters, 14% (95% CI, 11%-16%) were members of racial and ethnic minority groups (including but not limited to American Indian or Alaska Native, Asian, Black, Hispanic or Latinx, and Native Hawaiian or Pacific Islander), and 48% (95% CI, 44%-52%) were employed. Most participants supported work requirements generally (69% [95% CI, 66%-72%]) but did not support terminating benefits due to noncompliance (43% [95% CI, 39%-46%]) or requiring quotas of 20 or more hours per week (34% [95% CI, 31%-38%]). Support for monthly premiums (34% [95% CI, 31%-38%]) and exclusion penalties for premium nonpayment (22% [95% CI, 19%-25%]) was limited. Medicaid enrollees were significantly less supportive of these policies than nonenrollees. For instance, regarding work requirements, agreement was lower (64% [95% CI, 59%-69%] vs 72% [95% CI, 68%-77%]) and disagreement higher (26% [95% CI, 21%-31%] vs 20% [95% CI, 16%-24%]) among current Medicaid enrollees compared with nonenrollees (*P* = .04). Among Medicaid enrollees, some beliefs about work requirements varied significantly by employment status but not by political affiliation. Among nonenrollees, beliefs about work requirements, premiums, and Medicaid varied significantly by political affiliation but not by employment.

**Conclusions and Relevance:**

This study suggests that even when public constituencies express general support for Medicaid work requirements or premiums, they may oppose central design features, such as quotas and termination of benefits. Program participants may also hold significantly different beliefs than nonparticipants, which should be understood before policies are changed.

## Key Points

**Question:**

How do adult residents of Kentucky perceive general and specific policies regarding Medicaid work requirements and premiums, and do views differ by Medicaid enrollment?

**Findings:**

This survey study found that, despite general support for Medicaid work requirements, most respondents did not support terminating Medicaid benefits or setting quotas requiring 20 or more weekly hours, and fewer than half of respondents supported Medicaid premiums or terminating benefits to penalize nonpayment. Beliefs differed between Medicaid enrollees and nonenrollees.

**Meaning:**

This study suggests that public support may differ for general policy ideas vs specific policy elements and for people who are directly affected by a proposed policy change.

## Teaser

This survey study examines views of adults in Kentucky regarding the design of Medicaid work requirements and premiums.

## Introduction

Medicaid is vital to the US safety net, and it was a central means of federal support during the COVID-19 pandemic. As the national public health emergency concluded, more than 90 million people were enrolled in Medicaid and the Children’s Health Insurance Program.^1^ But as the public health emergency unwinds, political pressure to reduce Medicaid costs has escalated. Recent proposals—including a newly effective state waiver^2^ and debt negotiation proposals—have supported work requirements and premiums for adult Medicaid beneficiaries, particularly those eligible under the Patient Protection and Affordable Care Act (ACA) expansion.^3^

Repeated calls for Medicaid work requirements and premiums in state waivers, block grant proposals, and legislation suggest that these issues will persist.^4^ Work requirements were central to the Trump administration’s approach to public benefits^5^; the Centers for Medicare & Medicaid Services (CMS) approved work requirement waivers for 19 states during the Trump administration, asserting that they would increase health and reduce poverty.^6^ In practice, however, Medicaid work requirements were short-lived and likely harmful.^7,8,9^ In Arkansas—the only state that implemented work requirements with exclusion penalties during the Trump administration—nearly 18 000 people lost Medicaid, but employment and work hours were unchanged.^7^ In New Hampshire, which did not enforce penalties, 17 000 people (67% of eligible adults) fell short of required work hours in the first month.^10^

State Medicaid work requirements in Arkansas,^11^ New Hampshire,^12^ Kentucky,^13^ and Michigan^14^ were eventually struck down by federal courts, which found that CMS had failed to consider the projected coverage losses among beneficiaries. Other waivers were rescinded by the Biden administration, but a federal court reversed the cancellation of Georgia’s “Pathways to Coverage” program, which implemented work requirements and premiums this year.^15^ Rather than implementing a full ACA expansion, Georgia offers Medicaid to only “able-bodied” adults with eligible incomes who document 80 hours of qualifying activities per month.^16,17^ Arkansas has proposed a new waiver that offers more attractive Medicaid benefits to those who meet work engagement expectations.^18,19^

Federal work requirement proposals also continue, such as the Limit, Save, Grow Act of 2023 passed by the House of Representatives. This legislation would have omitted federal matching payments and allowed states to disenroll adult beneficiaries without 80 monthly hours of work or volunteering in 3 or more months.^20^ These initiatives echo work requirements in the Supplemental Nutrition Assistance Program and Temporary Assistance to Needy Families, which have been associated with losses of benefits that equal or exceed gains in earnings.^21,22^

Waivers requiring Medicaid beneficiaries to pay monthly premiums are ongoing. Georgia’s program, for example, requires premiums for new enrollees with incomes above 50% of the federal poverty level^16^; as of 2021, 8 other states had permission to charge monthly fees.^23^ Program evaluations have demonstrated that premiums reduce program enrollment,^24^ and the Biden administration has made efforts to rescind or limit waivers that enforce premiums.

Prior surveys have found support for Medicaid work requirements, ranging from 39% to 72% of respondents, but results vary by question phrasing and priming.^25,26,27^ Support has been higher among conservative people, those who view Medicaid as a short-term solution, those who believe that enrollees are undeserving, people not currently enrolled in Medicaid, older people, and non-Hispanic White individuals with greater racial resentment.^25,26,27,28^

General policy views, however, do not provide feedback about specific program features. Prior studies have reported general attitudes regarding work requirements (eg, “Do you favor or oppose requiring low-income, able-bodied adults without young children to work in order to receive Medicaid benefits?”^29,30^) but do not track views on specific policy choices. Although policymakers may anticipate that work requirements or premiums entail terminating benefits for noncompliance, the general public may not support this consequence. Describing Medicaid policies at a high level of generality may mask program features that, if enacted, would receive sharp opposition.

To inform Medicaid policy discussions, we assessed general views about work requirements and premiums, specific views about work-hour quotas, and specific views about terminating coverage to penalize noncompliance. We conducted the survey in Kentucky, which obtained the first Medicaid waiver allowing work requirements. Although the waiver was paused pending litigation and was later withdrawn, it would have required eligible beneficiaries to pay premiums and report 20 or more hours of weekly work or volunteering.

## Methods

We conducted an anonymous survey of Kentucky adults via the telephone and internet. We included noninstitutionalized Kentucky residents aged 18 years or older from June 27 through July 11, 2019. Surveys lasted a mean of 17 minutes and included demographic characteristics (eg, race and ethnicity, age, gender, and education), policy attitudes, and other beliefs about Medicaid. They were administered in English by trained staff of SSRS, an external survey research firm. Informed consent was obtained by telephone or internet before the questions began. The study protocol was approved by institutional review boards at the University of Pennsylvania, Columbia University, and SSRS. We report response information recommended by the American Association for Public Opinion Research (AAPOR) reporting guideline for cross-sectional surveys.^31^

Random-digit dialing of landlines and cell phones yielded 601 completed surveys. Of 39 110 landlines called, 209 reached an eligible person (of whom 150 completed the survey), 8654 were of unknown eligibility, and 30 247 were ineligible. Of 55 305 cell phone lines called, 617 reached an eligible person (of whom 451 completed the survey), 29 951 were of unknown eligibility, and 24 737 were ineligible. Response rates were 3.6% for landline and 5.2% for cell phone participants, using AAPOR definitions. Sampling for both types of telephone line divided the 120 Kentucky counties into 8 different strata based on the percentage of households with incomes below 138% of the federal poverty level. We oversampled from low-income strata to ensure sufficient sampling of Medicaid-eligible participants. We also oversampled cell phones flagged as prepaid and compensated these participants $10 for their minutes. SSRS interviewers conducted cell phone interviews with the person who answered, and landline interviews with the youngest adult home at the time of the call. All interviewers confirmed that the person was an adult and in a safe location before administering the survey.

The internet panel recruited 602 respondents from an invited, nonprobability panel of adult Kentucky residents maintained by Dynata. Sampling used the same county-based method as above. Respondents were excluded if they skipped 20% or more of the questions asked of all participants, failed at least 2 of 3 attention-check questions, or finished in less than 30% of the median survey time. Dynata compensated online respondents at standard rates.

Statistical analysis was performed from October 2019 to August 2023. Responses were weighted to represent the Kentucky adult population by age, gender, race and ethnicity, telephone use, poverty level, internet access, and geographical region. Base weights, adjusted for probability of selection, age, prepaid cell phone, and the strata listed above, were put through poststratification hot decking to balance the sample demographic profile to the 2017 American Community Survey sample of Kentucky adults. A calibration weight was added to reduce bias arising from different modes of survey administration.

We assessed participants’ agreement with Medicaid policy positions, using Likert scales from 1 (strongly disagree) to 7 (strongly agree). We classified 5, 6, or 7 as agree; 1, 2, or 3 as disagree; and 4 as neither agree nor disagree. The order of attitudinal questions was randomized, and questions drew on prior studies of Medicaid stigma.^32,33^ This study reports results for participants overall and by self-reported current enrollment in Medicaid. We further disaggregated each group by self-reported employment status and by political affiliation. For each pairwise group comparison, we used Pearson χ^2^ tests with the Rao and Scott second-order correction to compare distributions of beliefs (agree, neither, or disagree); this approach accounts for our sampling and weighting methods. All *P* values were from 2-sided tests and results were deemed statistically significant at *P* < .05.

## Results

Results in text and in Table 1, Table 2, Table 3, and Table 4 report weighted percentages, adjusted to reflect demographic characteristics of the Kentucky adult population. The demographic characteristics of the 1203 participants (52% women, 48% men; 80% younger than 65 years) differed by Medicaid enrollment (Table 1). Enrollees were more likely to be female, young, unmarried or separated, and members of racial and ethnic minority groups (including but not limited to American Indian or Alaska Native, Asian, Black, Hispanic or Latinx, and Native Hawaiian or Pacific Islander); they had less formal education and greater unemployment or disability compared with nonenrollees. There was little difference between enrollees and nonenrollees in political party affiliation, but enrollees were less likely to be registered to vote.

**Table 1.** Demographic Characteristics, by Percentages of Medicaid Enrollees vs Nonenrollees

Characteristic	No. (%) [95% CI for %]^a^			
	All (N = 1203)	By enrollment in Medicaid		
		Enrolled (n = 548)	Not enrolled (n = 633)	*P* value
Gender^b^^,^^c^				
Male	564 (47.7) [44.1-51.3]	216 (40.8) [35.5-46.4]	341 (53.3) [48.5-58.1]	.001
Female	633 (51.7) [48.1-55.3]	329 (58.8) [53.3-64.1]	290 (46.5) [41.7-51.3]	
Age, y^c^				
18-29	182 (19.7) [16.7-23.0]	109 (26.4) [21.5-32.0]	65 (13.7) [10.4-17.9]	<.001
30-49	376 (33.1) [29.8-36.7]	177 (28.1) [23.7-33.1]	191 (36.7) [32.0-41.7]	
50-64	347 (27.1) [24.1-30.3]	151 (25.2) [20.9-30.1]	193 (29.2) [25.1-33.7]	
≥65	289 (20.1) [17.6-22.9]	111 (20.2) [16.2-25.0]	175 (20.4) [17.2-24.0]	
Educational level^c^				
Less than high school	124 (13.1) [10.7-16.0]	96 (24.5) [19.7-29.9]	22 (4.3) [2.7-6.9]	<.001
Graduated high school	358 (33.1) [29.7-36.7]	194 (35.1) [30.1-40.6]	156 (31.3) [26.7-36.3]	
Vocational training, 2-y degree, or some college	382 (30.5) [27.3-33.9]	172 (27.0) [22.5-32.0]	206 (33.3) [28.8-38.1]	
4-y College or postgraduate degree	338 (23.3) [20.6-26.1]	86 (13.4) [10.4-17.2]	249 (31.1) [27.1-35.4]	
Race and ethnicity^c^				
Hispanic or Latinx	29 (2.5) [1.6-3.9]	12 (2.4) [1.1-4.9]	17 (2.6) [1.5-4.7]	.02
Non-Hispanic Black	87 (8.0) [6.1-10.3]	51 (11.0) [7.8-15.3]	33 (5.1) [3.4-7.7]	
Non-Hispanic White	1047 (86.5) [83.8-88.8]	466 (83.7) [79.1-87.5]	564 (89.1) [85.8-91.8]	
Other (non-Hispanic or Latinx)^d^	21 (1.3) [0.8-2.1]	10 (1.5) [0.7-2.9]	9 (1.0) [0.5-2.1]	
Sexual orientation^c^^,^^e^				
Lesbian, gay, bisexual, or something else	77 (6.5) [5.0-8.5]	44 (7.3) [5.0-10.4]	32 (6.1) [4.1-9.0]	.58
Straight	1095 (90.5) [88.2-92.5]	494 (91.3) [88.0-93.7]	583 (90.7) [87.3-93.2]	
Marital status^c^				
Married	594 (47.4) [43.8-51.0]	198 (30.4) [25.9-35.3]	386 (59.7) [54.8-64.4]	<.001
Never married	244 (22.7) [19.6-26.0]	147 (30.2) [25.3-35.7]	92 (17.5) [13.9-21.9]	
Divorced, widowed, or separated	302 (24.3) [21.3-27.5]	158 (30.7) [25.7-36.2]	138 (19.5) [16.1-23.4]	
Member of an unmarried couple	59 (5.3) [3.9-7.3]	44 (8.5) [5.8-12.3]	14 (2.7) [1.5-5.1]	
Registered to vote^c^				
Registered to vote	980 (80.0) [76.8-82.8]	417 (76.0) [70.9-80.4]	552 (84.1) [79.8-87.6]	.009
Not registered to vote	215 (19.3) [16.5-22.5]	128 (23.4) [19.0-28.4]	76 (15.2) [11.7-19.4]	
Political party^c^				
Republican	444 (35.7) [32.3-39.2]	187 (34.1) [29.1-39.5]	247 (36.0) [31.5-40.7]	.76
Democrat	375 (32.2) [28.9-35.6]	178 (33.7) [28.7-39.1]	193 (31.8) [27.4-36.4]	
Independent	224 (19.4) [16.6-22.5]	101 (18.3) [14.5-23.0]	120 (20.4) [16.6-24.8]	
Something else	134 (10.7) [8.7-13.1]	70 (11.6) [8.4-15.8]	60 (10.0) [7.5-13.3]	
Health insurance^f^				
Medicaid	548 (41.1) [37.6-44.7]	548 (100.0)	0	Not applicable
Medicare	415 (29.4) [26.4-32.7]	206 (37.3) [32.2-42.8]	203 (24.0) [20.4-27.9]	<.001
Employer sponsored	503 (49.1) [45.5-52.8]	109 (25.8) [21.0-31.3]	388 (66.8) [62.2-71.1]	<.001
Purchased directly	259 (22.3) [19.4-25.6]	122 (27.3) [22.3-32.8]	133 (19.2) [15.7-23.3]	.008
TRICARE, Veterans Affairs, or other military	84 (7.3) [5.6-9.4]	34 (6.9) [4.6-10.2]	50 (7.9) [5.6-11.0]	.67
Other	66 (5.5) [4.1-7.4]	24 (4.2) [2.6-6.8]	41 (6.6) [4.5-9.5]	.17
Uninsured	79 (6.6) [5.3-8.1]	0	71 (11.2) [9.0-13.9]	<.001
Employed				
Yes	510 (47.8) [44.3-51.5]	155 (31.7) [26.7-37.1]	345 (59.0) [54.2-63.7]	<.001
No	222 (17.6) [15.0-20.5]	153 (25.9) [21.5-30.9]	64 (11.8) [8.8-15.6]	
Disabled	184 (13.0) [10.9-15.6]	107 (22.6) [18.3-27.5]	46 (6.0) [4.1-8.6]	
Retired	287 (21.5) [18.8-24.5]	133 (19.9) [15.9-24.5]	178 (23.2) [19.6-27.3]	
Hours worked last week, if employed^g^				
<20	38 (6.6) [4.5-9.7]	22 (13.3) [7.8-21.9]	15 (3.9) [2.2-7.0]	.001
≥20	472 (93.4) [90.3-95.5]	133 (86.7) [78.1-92.2]	330 (96.1) [93.0-97.8]	

^a^ Percentages are weighted to resemble adult population of Kentucky residents.

^b^ Excludes 2 participants who identify as “other or different gender identity.”

^c^ Percentages may not add to 100% because of “declined,” “don’t know,” or “refused” responses (80% of respondents [95% CI 77%-82%] reported ages <65 years).

^d^ Included American Indian or Alaska Native, Asian, Native Hawaiian or Pacific Islander, and a write-in field where participants could supply their own race and/or ethnicity (14% of respondents [95% CI, 11%-16%] reported being members of racial and ethnic minority groups [a category other than non-Hispanic White]).

^e^ Response categories for sexual orientation included lesbian or gay, straight (this term was used instead of heterosexual), bisexual, and something else.

^f^ Participants could select multiple sources of current insurance coverage, so totals across all categories exceed 100%. The “uninsured” response category includes participants who answered “no,” “declined,” “don’t know,” or “refused” to every source of health insurance named.

^g^ Among respondents who indicated they were employed (510 participants in full sample; 345 people not enrolled in Medicaid; 155 people enrolled in Medicaid).

**Table 2.** Views of Medicaid Policy, by Medicaid Enrollment

Views	No. (%) [95% CI for %]^a^			
	All (N = 1203)	By enrollment in Medicaid		
		Enrolled (n = 548)	Not enrolled (n = 633)	*P* value
Work requirement attitudes^b^				
“If people are able, they should be required to spend time volunteering or working to stay on Medicaid.”				
Agree	767 (69.0) [65.7-72.2]	320 (64.0) [58.7-69.0]	435 (72.3) [68.2-76.5]	.04
Disagree	253 (22.2) [19.3-25.3]	129 (25.6) [21.0-30.8]	119 (19.8) [16.2-24.0]	
Neither agree nor disagree	181 (8.6) [7.2-10.3]	97 (9.9) [7.6-12.8]	79 (7.7) [5.9-9.9]	
“If people who have Medicaid are required to spend time volunteering or working, but they do not complete the requirement, they should lose their Medicaid benefits.”				
Agree	487 (42.7) [39.1-46.3]	182 (35.5) [30.4-40.9]	298 (47.6) [42.8-52.4]	.004
Disagree	471 (39.8) [36.4-43.4]	245 (45.9) [40.5-51.4]	218 (36.0) [31.4-40.8]	
Neither agree nor disagree	236 (17.0) [14.5-19.8]	118 (18.0) [14.1-22.6]	112 (16.1) [13.0-19.8]	
Preferred weekly work quota, h^b^				
0	178 (13.3) [11.2-15.7]	84 (14.5) [11.1-18.7]	89 (11.9) [9.4-15.0]	.11
1-19	593 (50.8) [47.2-54.4]	292 (53.3) [47.8-58.7]	292 (49.2) [44.4-54.1]	
20	250 (20.5) [17.8-23.5]	107 (19.2) [15.3-23.8]	138 (21.7) [18.0-25.8]	
21-39	98 (8.0) [6.3-10.1]	38 (7.7) [5.2-11.4]	59 (8.4) [6.2-11.3]	
≥40	57 (5.2) [3.7-7.2]	16 (3.0) [1.7-5.1]	40 (6.7) [4.4-10.0]	
Mandatory premium attitudes^b^				
“People should be required to pay some money out of pocket each month to have Medicaid.”				
Agree	406 (34.3) [31.0-37.8]	148 (28.2) [23.6-33.4]	251 (38.2) [33.7-43.0]	.004
Disagree	515 (49.7) [46.1-53.3]	271 (56.7) [51.3-62.0]	236 (45.5) [40.7-50.4]	
Neither agree nor disagree	271 (15.1) [13.1-17.4]	126 (14.5) [11.5-18.1]	139 (15.3) [12.6-18.5]	
“If people who have Medicaid are required to pay money each month, but they miss payments, they should lose their Medicaid benefits.”				
Agree	241 (21.5) [18.6-24.6]	86 (19.9) [15.6-25.1]	152 (22.5) [18.8-26.8]	.20
Disagree	740 (62.1) [58.5-65.5]	370 (66.1) [60.6-71.2]	361 (59.9) [55.1-64.5]	
Neither agree nor disagree	214 (16.0) [13.6-18.7]	89 (13.4) [10.1-17.6]	116 (17.2) [13.9-21.0]	
General Medicaid attitudes^b^				
“In general, people are on Medicaid due to circumstances beyond their control.”				
Agree	723 (59.7) [56.1-63.2]	379 (69.0) [63.7-73.8]	335 (54.0) [49.1-58.7]	<.001
Disagree	230 (18.8) [16.2-21.7]	66 (11.1) [8.3-14.7]	163 (24.5) [20.6-28.8]	
Neither agree nor disagree	240 (20.4) [17.6-23.5]	100 (18.8) [14.8-23.6]	129 (20.3) [16.7-24.5]	
“In general, society has a responsibility to help people who are on Medicaid.”				
Agree	578 (49.2) [45.6-52.8]	280 (53.4) [47.9-58.8]	290 (46.8) [42.0-51.6]	.15
Disagree	321 (27.4) [24.3-30.7]	121 (24.1) [19.6-29.2]	196 (29.7) [25.5-34.3]	
Neither agree nor disagree	292 (22.4) [19.6-25.6]	142 (21.5) [17.4-26.2]	140 (22.5) [18.7-26.8]	
“Medicaid should only be for people who cannot work, like children, disabled people, and the elderly.”				
Agree	678 (60.3) [56.8-63.7]	255 (51.4) [45.9-56.9]	411 (66.2) [61.6-70.6]	<.001
Disagree	351 (28.2) [25.2-31.5]	203 (35.4) [30.5-40.7]	143 (23.5) [19.6-27.9]	
Neither agree nor disagree	168 (11.2) [9.2-13.5]	89 (13.0) [9.8-17.1]	75 (9.9) [7.6-12.9]	
“For people who are able to work, being on Medicaid makes them less interested in working.”				
Agree	579 (49.6) [46.0-53.2]	224 (43.8) [38.4-49.4]	345 (53.9) [49.1-58.7]	.02
Disagree	433 (35.8) [32.4-39.3]	226 (39.5) [34.3-45.0]	201 (33.4) [29.0-38.1]	
Neither agree nor disagree	184 (14.0) [11.7-16.7]	94 (16.0) [12.4-20.4]	84 (12.2) [9.4-15.7]	
Perceived proportion of Medicaid enrollees who receive but do not need benefits, %^b^				
≤25	442 (35.3) [32.0-38.8]	209 (34.7) [29.8-39.9]	224 (35.8) [31.3-40.5]	.84
26-50	422 (34.3) [31.0-37.8]	200 (36.0) [30.9-41.4]	217 (33.9) [29.5-38.6]	
51-75	221 (19.5) [16.6-22.7]	86 (17.9) [13.8-23.0]	131 (20.5) [16.7-24.8]	
76-100	97 (9.2) [7.2-11.6]	43 (8.8) [6.1-12.5]	50 (8.7) [6.2-12.0]	

^a^ Percentages are weighted to resemble the adult population of Kentucky residents.

^b^ Percentages may not add to 100% because of “declined,” “don’t know,” or “refused” responses.

**Table 3.** Views of Medicaid Policy Among Medicaid Enrollees, by Employment and Political Affiliation

Views	Medicaid enrollees by employment^a^			Medicaid enrollees by political affiliation^b^		
	Employed, No. (%) [95% CI for %] (n = 155)^c^	Unemployed, No. (%) [95% CI for %] (n = 153)^c^	*P* value	Republican, No. (%) [95% CI for %] (n = 187)^c^	Democrat, No. (%) [95% CI for %] (n = 178)^c^	*P* value
Work requirement attitudes^d^						
“If people are able, they should be required to spend time volunteering or working to stay on Medicaid.”						
Agree	94 (68.2) [58.4-76.6]	70 (52.7) [42.2-62.9]	.005	122 (67.2) [57.8-75.5]	103 (67.5) [58.8-75.2]	.87
Disagree	27 (16.9) [10.4-26.2]	51 (36.9) [27.5-47.4]		32 (22.0) [14.7-31.6]	49 (23.1) [16.7-31.1]	
Neither agree nor disagree	33 (14.0) [9.1-21.0]	32 (10.4) [6.5-16.4]		32 (10.3) [6.4-16.1]	25 (8.5) [5.0-14.1]	
“If people who have Medicaid are required to spend time volunteering or working, but they do not complete the requirement, they should lose their Medicaid benefits.”						
Agree	54 (36.0) [26.9-46.3]	36 (25.5) [17.1-36.0]	.28	74 (46.1) [37.0-55.5]	57 (33.5) [25.2-43.0]	.13
Disagree	65 (41.0) [31.5-51.3]	78 (51.3) [40.8-61.7]		71 (36.8) [28.5-45.9]	89 (48.9) [39.4-58.4]	
Neither agree nor disagree	35 (22.0) [14.5-32.1]	39 (23.2) [15.2-33.8]		42 (17.1) [11.5-24.8]	30 (16.4) [10.1-25.6]	
Preferred weekly work quota, h^d^						
0	14 (7.1) [3.7-13.3]	38 (21.9) [15.0-30.7]	.003	19 (11.3) [6.6-18.8]	33 (13.2) [8.7-19.6]	.79
1-19	85 (58.9) [48.8-68.3]	86 (57.5) [47.0-67.4]		103 (49.1) [39.9-58.3]	94 (58.1) [48.6-67.1]	
20	33 (19.1) [12.8-27.6]	20 (13.5) [7.7-22.8]		35 (20.5) [14.0-28.9]	35 (18.0) [12.0-26.1]	
21-39	16 (12.1) [6.7-20.7]	5 (2.4) [0.9-6.5]		16 (9.5) [5.1-17.1]	10 (7.7) [3.5-16.4]	
≥40	6 (2.6) [1.1-6.1]	2 (2.7) [0.7-10.2]		7 (4.1) [1.8-9.2]	4 (2.5) [0.9-7.2]	
Mandatory premium attitudes^d^						
“People should be required to pay some money out of pocket each month to have Medicaid.”						
Agree	53 (32.6) [24.1-42.5]	30 (24.1) [15.7-35.1]	.42	66 (35.6) [27.4-44.8]	49 (27.2) [19.4-36.7]	.13
Disagree	63 (51.2) [41.1-61.2]	81 (56.8) [46.1-66.9]		78 (48.7) [39.5-58.0]	97 (61.8) [52.3-70.5]	
Neither agree nor disagree	39 (16.2) [10.8-23.6]	42 (19.1) [12.5-28.2]		41 (15.1) [10.0-22.1]	32 (11.0) [7.2-16.6]	
“If people who have Medicaid are required to pay money each month, but they miss payments, they should lose their Medicaid benefits.”						
Agree	33 (25.8) [17.5-36.4]	17 (12.2) [6.7-21.0]	.07	31 (18.2) [11.9-26.8]	30 (25.5) [17.2-36.1]	.21
Disagree	96 (57.3) [46.8-67.2]	107 (71.4) [60.8-80.1]		119 (62.7) [53.0-71.4]	124 (62.6) [52.4-71.7]	
Neither agree nor disagree	25 (15.9) [9.4-25.7]	29 (16.5) [9.9-26.2]		35 (18.5) [11.8-27.8]	23 (11.1) [6.5-18.1]	
General Medicaid attitudes^d^						
“In general, people are on Medicaid due to circumstances beyond their control.”						
Agree	106 (66.0) [55.6-75.1]	99 (64.3) [53.7-73.7]	.32	120 (61.6) [52.1-70.2]	139 (77.5) [68.5-84.5]	.02
Disagree	17 (12.2) [6.9-20.8]	14 (7.7) [4.2-13.7]		32 (17.7) [11.7-25.8]	16 (7.3) [4.1-12.6]	
Neither agree nor disagree	30 (19.9) [12.7-29.8]	40 (28.0) [19.3-38.7]		33 (18.5) [12.2-27.1]	22 (14.3) [8.5-23.2]	
“In general, society has a responsibility to help people who are on Medicaid.”						
Agree	94 (57.8) [47.3-67.6]	65 (47.3) [37.0-57.9]	.39	89 (55.6) [46.5-64.4]	107 (57.5) [47.7-66.8]	.36
Disagree	27 (18.4) [11.9-27.4]	31 (22.8) [14.9-33.3]		46 (24.7) [17.7-33.4]	33 (18.7) [12.4-27.2]	
Neither agree nor disagree	33 (23.6) [15.5-34.3]	56 (29.6) [21.3-39.4]		49 (17.6) [12.6-24.1]	38 (23.8) [16.1-33.7]	
“Medicaid should only be for people who cannot work, like children, disabled people, and the elderly.”						
Agree	68 (49.2) [39.1-59.3]	40 (30.0) [21.1-40.8]	.02	103 (63.9) [55.1-71.9]	84 (52.7) [43.2-62.1]	.12
Disagree	60 (36.7) [27.8-46.7]	78 (53.2) [42.6-63.5]		60 (27.9) [20.8-36.2]	64 (33.2) [25.2-42.3]	
Neither agree nor disagree	27 (14.1) [8.6-22.3]	35 (16.8) [10.6-25.5]		23 (7.7) [4.3-13.4]	30 (14.1) [8.7-22.0]	
“For people who are able to work, being on Medicaid makes them less interested in working.”						
Agree	57 (41.2) [31.5-51.6]	51 (37.0) [27.2-48.1]	.80	97 (55.6) [46.4-64.5]	60 (38.9) [29.8-48.8]	.05
Disagree	65 (39.1) [29.7-49.3]	70 (43.5) [33.6-54.1]		59 (28.7) [21.2-37.6]	84 (41.3) [32.4-50.8]	
Neither agree nor disagree	32 (18.8) [12.2-27.8]	32 (19.4) [12.7-28.6]		29 (15.1) [9.8-22.6]	33 (19.0) [12.3-28.2]	
Perceived proportion of Medicaid enrollees who receive but do not need benefits, %^d^						
≤25	44 (28.4) [20.0-38.5]	74 (46.1) [36.0-56.6]	.12	60 (30.7) [22.9-39.9]	84 (40.7) [32.0-50.1]	.30
26-50	63 (38.7) [29.4-48.9]	43 (29.9) [20.8-41.0]		76 (39.6) [31.1-48.8]	56 (27.9) [20.4-36.9]	
51-75	30 (21.4) [13.9-31.5]	23 (15.5) [9.1-25.3]		31 (19.1) [12.2-28.6]	26 (20.3) [12.8-30.8]	
76-100	16 (9.0) [5.1-15.4]	12 (8.1) [3.9-16.0]		18 (9.9) [5.9-16.2]	9 (8.4) [4.2-16.4]	

^a^ Excludes individuals on Medicaid who reported being disabled (n = 133) or retired (n = 107).

^b^ For responses to the question “In politics today, do you consider yourself…,” individuals choosing Independent (n = 101), something else (n = 70), or don’t know or refused (n = 12) were excluded from these analyses.

^c^ Percentages are weighted to resemble the adult population of Kentucky residents.

^d^ Percentages may not add to 100% because of “declined,” “don’t know,” or “refused” responses.

**Table 4.** Views of Medicaid Policy Among Medicaid Nonenrollees, by Employment and Political Affiliation

Views	Medicaid nonenrollees by employment^a^			Medicaid nonenrollees by political affiliation^b^		
	Employed, No. (%) [95% CI for %] (n = 345)^c^	Unemployed, No. (%) [95% CI for %] (n = 64)^c^	*P* value	Republican, No. (%) [95% CI for %] (n = 247)^c^	Democrat, No. (%) [95% CI for %] (n = 193)^c^	*P* value
Work requirement attitudes^d^						
“If people are able, they should be required to spend time volunteering or working to stay on Medicaid.”						
Agree	232 (70.7) [64.8-76.1]	43 (74.0) [59.3-84.7]	.88	197 (80.3) [72.8-86.2]	106 (60.1) [51.7-67.9]	<.001
Disagree	67 (21.1) [16.3-26.9]	13 (18.3) [9.4-32.5]		29 (14.4) [9.2-22.0]	58 (30.4) [23.3-38.6]	
Neither agree nor disagree	46 (8.1) [5.8-11.3]	8 (7.8) [3.1-18.2]		21 (5.3) [3.0-9.1]	29 (9.5) [6.2-14.3]	
“If people who have Medicaid are required to spend time volunteering or working, but they do not complete the requirement, they should lose their Medicaid benefits.”						
Agree	150 (44.1) [37.8-50.6]	27 (46.6) [31.6-62.3]	.95	144 (58.2) [50.3-65.8]	66 (35.2) [27.4-44.0]	<.001
Disagree	134 (40.2) [34.1-46.7]	21 (37.8) [23.8-54.1]		60 (24.3) [18.1-31.8]	97 (51.4) [42.8-59.9]	
Neither agree nor disagree	59 (15.5) [11.5-20.5]	15 (15.3) [7.4-29.1]		40 (16.9) [11.8-23.6]	30 (13.4) [8.6-20.1]	
Preferred weekly work quota, h^d^						
0	48 (13.1) [9.6-17.6]	12 (13.4) [6.8-24.8]	.94	14 (3.9) [2.1-7.1]	49 (23.4) [17.3-30.8]	<.001
1-19	154 (47.5) [41.1-54.0]	29 (51.8) [36.3-66.9]		111 (50.4) [42.6-58.2]	88 (45.1) [36.6-53.8]	
20	88 (24.5) [19.5-30.4]	13 (19.1) [9.6-34.4]		72 (26.8) [20.7-33.9]	27 (16.3) [10.7-24.0]	
21-39	30 (6.9) [4.5-10.6]	6 (8.4) [3.2-20.4]		25 (10.4) [6.4-16.6]	16 (7.1) [4.1-12.1]	
≥40	17 (5.7) [3.1-10.3]	3 (6.3) [1.4-24.1]		17 (6.5) [3.5-11.9]	10 (5.8) [2.6-12.2]	
Mandatory premium attitudes^d^						
“People should be required to pay some money out of pocket each month to have Medicaid.”						
Agree	135 (35.5) [29.7-41.8]	19 (32.0) [19.5-47.8]	.80	116 (43.7) [36.2-51.5]	70 (35.2) [27.5-43.8]	.06
Disagree	132 (47.4) [40.9-53.9]	21 (48.1) [32.8-63.8]		74 (36.9) [29.3-45.1]	80 (50.2) [41.7-58.8]	
Neither agree nor disagree	75 (16.5) [12.8-21.1]	24 (19.8) [11.8-31.4]		54 (18.4) [13.4-24.7]	42 (13.7) [9.7-19.1]	
“If people who have Medicaid are required to pay money each month, but they miss payments, they should lose their Medicaid benefits.”						
Agree	81 (20.1) [15.6-25.6]	17 (30.2) [17.8-46.3]	.11	80 (29.4) [22.9-36.8]	31 (17.7) [11.7-25.8]	.004
Disagree	203 (63.1) [56.8-69.0]	28 (46.2) [31.1-62.0]		113 (51.0) [43.2-58.8]	131 (70.2) [61.9-77.4]	
Neither agree nor disagree	59 (16.4) [12.3-21.5]	19 (23.7) [13.3-38.7]		52 (19.3) [14.1-25.8]	31 (12.1) [8.0-17.8]	
General Medicaid attitudes^d^						
“In general, people are on Medicaid due to circumstances beyond their control.”						
Agree	185 (57.1) [50.6-63.3]	25 (40.1) [25.9-56.3]	.16	110 (46.1) [38.4-54.0]	124 (62.9) [54.1-70.9]	.01
Disagree	96 (25.0) [19.9-30.9]	17 (29.1) [16.9-45.2]		87 (32.8) [26.1-40.4]	36 (21.1) [14.6-29.4]	
Neither agree nor disagree	62 (17.4) [13.1-22.8]	21 (26.2) [15.3-41.2]		50 (21.1) [15.3-28.3]	31 (14.5) [9.5-21.6]	
“In general, society has a responsibility to help people who are on Medicaid.”						
Agree	170 (50.2) [43.7-56.6]	17 (30.3) [17.5-47.1]	.06	90 (36.5) [29.3-44.3]	116 (58.7) [49.9-66.9]	<.001
Disagree	98 (26.2) [21.1-32.2]	23 (38.4) [24.4-54.6]		103 (39.0) [31.8-46.8]	38 (23.4) [16.6-32.1]	
Neither agree nor disagree	74 (22.6) [17.5-28.6]	24 (31.4) [19.2-46.8]		52 (24.3) [17.8-32.2]	36 (15.9) [10.7-22.9]	
“Medicaid should only be for people who cannot work, like children, disabled people, and the elderly.”						
Agree	207 (61.2) [54.8-67.3]	38 (59.1) [43.1-73.4]	.84	192 (81.1) [74.6-86.2]	103 (54.7) [46.0-63.0]	<.001
Disagree	95 (27.9) [22.4-34.0]	14 (27.1) [15.0-44.0]		30 (9.7) [6.4-14.4]	66 (36.6) [28.7-45.3]	
Neither agree nor disagree	42 (10.8) [7.6-15.1]	12 (13.8) [6.6-26.5]		23 (8.8) [5.2-14.6]	23 (8.3) [5.1-13.2]	
“For people who are able to work, being on Medicaid makes them less interested in working.”						
Agree	177 (50.0) [43.6-56.5]	28 (43.9) [29.2-59.8]	.52	171 (68.5) [60.7-75.4]	86 (46.3) [37.8-55.0]	.001
Disagree	119 (35.8) [29.8-42.2]	20 (36.0) [22.3-52.4]		50 (21.9) [16.1-29.2]	80 (39.7) [31.8-48.2]	
Neither agree nor disagree	47 (13.6) [9.7-18.7]	16 (20.1) [10.8-34.5]		25 (9.4) [5.7-15.2]	25 (12.6) [7.7-19.9]	
Perceived proportion of Medicaid enrollees who receive but do not need benefits, %^d^						
≤25	118 (36.2) [30.3-42.6]	25 (33.8) [20.6-50.1]	.77	62 (23.6) [17.5-31.0]	90 (47.6) [39.1-56.2]	<.001
26-50	119 (34.5) [28.7-40.9]	22 (34.5) [21.7-50.0]		96 (41.8) [34.3-49.8]	61 (29.6) [22.5-37.9]	
51-75	74 (19.5) [14.8-25.2]	13 (25.8) [13.7-43.1]		61 (22.2) [16.5-29.1]	31 (16.6) [10.7-24.9]	
76-100	29 (8.8) [5.6-13.4]	3 (5.6) [1.6-17.9]		26 (11.8) [7.4-18.2]	8 (4.8) [2.0-11.2]	

^a^ Excludes individuals not enrolled in Medicaid who reported being disabled (n = 46) or retired (n = 178).

^b^ For responses to the question “In politics today, do you consider yourself…,” individuals choosing Independent (n = 120), something else (n = 60), or don’t know or refused (n = 13) were excluded from these analyses.

^c^ Percentages are weighted to resemble the adult population of Kentucky residents.

^d^ Percentages may not add to 100% because of “declined,” “don’t know, ” or “refused” responses.

### Work Requirements

Most participants (69% [95% CI, 66%-72%]) agreed that “If people are able, they should be required to spend time volunteering or working to stay on Medicaid,” while 22% (95% CI, 19%-25%) disagreed (Figure and Table 2). Agreement was lower (64% [95% CI, 59%-69%] vs 72% [95% CI, 68%-77%]) and disagreement higher (26% [95% CI, 21%-31%] vs 20% [95% CI, 16%-24%]) among current Medicaid enrollees compared with nonenrollees (*P* = .04). When asked how many weekly hours of work or volunteering should be required, the modal response was in the range of 1 to 19 hours (Figure); a majority (64% [95% CI, 61%-71%]) endorsed weekly quotas below 20 hours.

**Figure.**
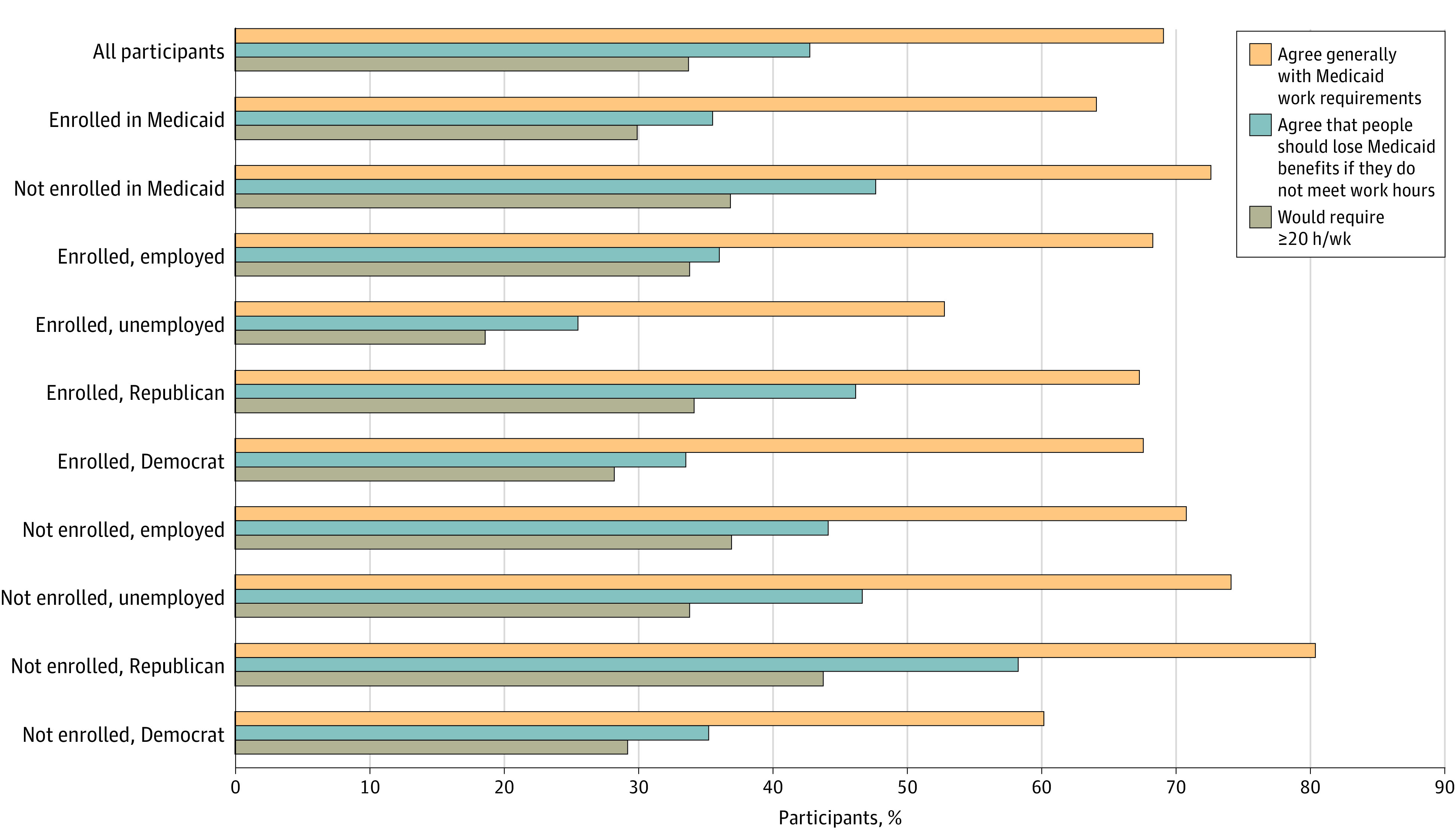
Views of Work Requirement Policy by Medicaid Enrollment, Employment, and Political Affiliation

Despite general support for work requirements, most participants did not support terminating benefits due to noncompliance (Figure and Table 2). Among the full sample, only 43% of participants (95% CI, 39%-46%) agreed that people with unmet hours “should lose their Medicaid benefits,” while 17% (95% CI, 15%-20%) took neither position and 40% (95% CI, 36%-43%) disagreed. Distributions of support, neutrality, and opposition varied significantly by Medicaid enrollment, with enrollees reporting greater disagreement (46% [95% CI, 41%-51%] vs 36% [95% CI, 31%-41%]) and less agreement (36% [95% CI; 30%-41%] vs 48% [95% CI, 43%-52%]) than nonenrollees (*P* = .004).

Among Medicaid enrollees (Table 3), the distribution of support for work requirements, exclusion penalties, and hour quotas did not differ by political affiliation. But several of these beliefs differed by employment, with employed enrollees more likely to support work requirements and higher quotas. However, support for benefits termination did not differ or reach a majority among any subgroup of Medicaid enrollees.

Among nonenrollees (Table 4), work requirement attitudes did not differ by employment status, but they differed significantly by political affiliation. Republican nonenrollees were more likely than Democratic nonenrollees to support and less likely to oppose work requirements and exclusion penalties, and they preferred higher quotas of work hours.

### Premiums

Most participants did not support monthly Medicaid premiums, with only 34% of participants (95% CI, 31%-38%), 28% of Medicaid enrollees (95% CI, 24%-33%), and 38% of nonenrollees (95% CI, 34%-43%) agreeing that “people should be required to pay some money out of pocket each month to have Medicaid” (Table 2). Enrollees reported less agreement and greater disagreement than nonenrollees. Support for enforcing premiums by benefits termination was low, at 22% overall (95% CI, 19%-25%), 20% of Medicaid enrollees (95% CI, 16%-25%), and 23% of nonenrollees (95% CI, 19%-27%). No subgroup reported majority support for either premiums or exclusion penalties.

Among Medicaid enrollees (Table 3), the distribution of support for premiums and for terminating nonpayers’ benefits did not differ by employment status or political affiliation. Among Medicaid nonenrollees (Table 4), beliefs about premiums did not differ by employment status, but again differed by political ideology; Democratic nonenrollees were less likely to support and more likely to oppose premiums and termination of benefits for nonpayment than Republican nonenrollees.

### Beliefs About Medicaid and Medicaid Enrollees

We also queried participants’ attitudes about Medicaid beneficiaries and the purposes of the program, and enrollees reported more favorable views than nonenrolles (Table 2). In the full sample, 60% of participants (95% CI, 56%-63%) agreed and 19% (95% CI, 16%-22%) disagreed that people are “on Medicaid due to circumstances beyond their control”; enrollees were significantly more likely to agree (69% [95% CI, 64%-74%] vs 54% [95% CI, 49%-59%]) and less likely to disagree (11% [95% CI, 8%-15%] vs 25% [95% CI, 21%-29%]) with the statement compared with nonenrollees. Among the entire sample, 60% of participants (95% CI, 57%-64%), as well as 51% of enrollees (95% CI, 46%-57%) and 66% of nonenrollees (95% CI, 62%-71%), believed that “Medicaid should only be for people who cannot work,” and enrollees were more likely than nonenrollees to disagree with this statement (35% [95% CI, 31%-41%] vs 24% [20%-28%]). Views also split significantly on whether Medicaid reduces beneficiaries’ interest in working, with agreement lower (44% [95% CI, 38%-49%] vs 54% [95% CI, 49%-59%]) and disagreement higher (40% [95% CI, 34%-45%] vs 33% [95% CI, 29%-38%]) among enrollees compared with nonenrollees. Overall, 49% of participants (95% CI, 46%-53%) believed that “society has a responsibility to help people who are on Medicaid,” and the distribution of this attitude did not differ across enrollment status. Most participants in the full sample, enrollees, and nonenrollees estimated that more than one-fourth of enrollees receive unneeded benefits; and the distribution of these beliefs did not differ by Medicaid enrollment.

Among Medicaid enrollees (Table 3), Democratic enrollees were more likely than Republican enrollees to agree that people are on Medicaid due to circumstances beyond their control. Beliefs about whether Medicaid reduces enrollees’ interest in working differed with marginal statistical significance, with Democratic enrollees less likely to agree and more likely to be neutral compared with Republican enrollees. Enrollees’ beliefs did not differ by employment status, except for the belief that “Medicaid should only be for people who cannot work,” which was endorsed by more employed enrollees compared with unemployed enrollees.

Among Medicaid nonenrollees (Table 4), political affiliation (but not employment status) was associated with significantly different beliefs about Medicaid beneficiaries. Republican nonenrollees were more likely to endorse the beliefs that Medicaid “should only be for people who cannot work” and that the program makes people “less interested in working”; they were less likely to endorse the ideas that people are on Medicaid due to circumstances beyond their control and that society has a responsibility to help them. Republican nonenrollees also believed that larger proportions of people “receive but do not need” Medicaid benefits.

## Discussion

In Kentucky, a state that had federal approval to implement work requirements and premiums for Medicaid, most participants expressed general agreement with work requirements for Medicaid enrollees who are able to volunteer or work. But only a minority of participants—among the full sample, those enrolled, and those not enrolled in Medicaid—supported the policy features that had been approved in their state, including termination of benefits for noncompliance and hourly work quotas of 20 hours or more. Among Medicaid enrollees, support was lower among people who were unemployed compared with those who were employed; among nonenrollees, support was lower among Democratic participants than among Republican particpants. Republican nonenrollees were the only subgroup to report majority support for terminating benefits for people who do not meet work or volunteer quotas.

Although monthly premiums have a longer history in Medicaid, they were uniformly less popular than work requirements across all groups. Support for mandatory premiums was lower among enrollees than nonenrollees, but these 2 subgroups reported uniform opposition to terminating benefits due to premium nonpayment. Among nonenrollees, Democratic participants reported less support for mandatory premiums and exclusion penalties than Republican participants, but no subgroup reported majority support for these policy components.

General views of Medicaid enrollees and purposes differed by Medicaid enrollment and political affiliation. Enrollees and Democratic participants reported more supportive views of Medicaid participants, and they estimated that lower percentages of enrollees “receive but do not need” Medicaid benefits, compared with nonenrollees and Republican participants. For the most part, employment status was not associated with differences in views about Medicaid’s beneficiaries and purposes, save for one finding: employed enrollees were more likely to endorse the belief that the program should be reserved “for people who cannot work.” This finding was surprising, given that employed enrollees personally benefited from the extension of Medicaid to working people. However, this view may coincide with other beliefs not measured here (eg, that employers should provide affordable health insurance that makes Medicaid unnecessary).

Our findings suggest that survey participants who support work requirements and premiums for Medicaid, in general, may not support specific policy features, such as terminating benefits for people who do not comply. People may envision and prefer enforcement mechanisms that do not affect benefits eligibility. They may also prefer smaller quotas than the 20-hour weekly threshold that has been repeatedly proposed.

Public preferences should influence state and federal policymakers considering benefits program design. People who depend on Medicaid for health care access have an intense interest in seeing their experiences reflected in program policies, given the high personal stakes.^34,35,36,37,38,39,40,41,42,43^ But even people who do not currently depend on Medicaid for health care access have a stake in whether elected politicians and regulators are acting with democratic legitimacy—that is, whether they are responding to voters’ priorities. Policies lacking popular backing are susceptible to litigation and political upheaval, as well as threats to program participation and compliance. Lawmakers’ responsiveness to public priorities can also affect participation in the political process. Research on social effects of Medicaid policymaking, for example, has shown that expansions of Medicaid benefits are associated with increased social and political engagement.^42,44,45,46^ Policymaking processes sometimes build in avenues for public input, such as notice-and-comment periods or town halls. But although these are important sources of information, participants in these venues may not be representative of the populations affected by policy change.^47,48^ Public comment periods also occur after extensive policy development and thus may be limited in their capacity to achieve change.^49^ Systematic and representative investigation of public views is an important complement to public comment procedures.

Responsiveness to the public is an important policy priority, but it is not the only consideration. Legislators and regulators must also consider governmental resources, urgency, feasibility, sustainability, predicted effectiveness, political ideology, and concerns such as fairness and equity. Deferring to a voting majority can also be associated with disadvantages for members of smaller or politically marginalized groups (eg, members of racial and ethnic minority groups), presenting equity concerns. Public preferences can also reflect invidious forces, and views of benefits often correspond to racial biases.^28^ To counteract these concerns, demographic differences in public opinion should also be of interest to policymakers. Even when policymakers are responding to reasons separate from public preferences (eg, political strategy and budget constraints), understanding the nuances of public opinion matters.

Responsiveness to public opinion also raises opportunities for research. Surveys of public views on work requirements, premiums, and other Medicaid policies should query not only general support but also views about specific policy features. In this sample, many participants supported work requirements but disagreed with enforcement mechanisms. Recent survey research has also found public preferences for work supports, such as childcare and transportation,^28^ that are linked to increased earnings.^21^ These findings suggest possible support for Medicaid programming that connects enrollees with childcare, transportation, tuition, or other benefits that facilitate labor force participation, delivered in a way that does not put benefits at risk. Mandatory premiums of any design—but particularly those accompanied by exclusion penalties—had limited support in this sample.

### Limitations

This study has several limitations. Data are self-reported and subject to social desirability bias. Post hoc analyses by survey mode showed that telephone participants reported greater agreement than internet participants for all Likert scale items, suggesting that the direction of social desirability bias may overstate agreement with work requirements and benefits termination penalties. Question wording was likely associated with responses despite our efforts to phrase prompts neutrally. Telephone survey response rates were low; rates for national polls are around 6% to 7%.^50,51^ We weighted responses to improve representativeness. Policy views may change under conditions of actual implementation, particularly among enrollees. Data were collected before COVID-19, which increased the proportion of the population enrolled in Medicaid (from 26% in 2019 to 30% in 2021), shifted portions of the labor market (eg, layoffs, telework, and rehiring), and increased federal support to states and families. In surveys conducted by the KFF Health Tracking Poll, however, public views of Medicaid were constant through COVID-19—including program approval (2019 vs 2023) and views on whether Medicaid is health insurance or welfare (2017 vs 2023).^52^

## Conclusions

In this survey study, we found that the preferences of Medicaid enrollees, nonenrollees, and the general adult population may depend not only on general policies (eg, work requirements and premiums) but also on specific policy features, such as penalties. Despite general support for work requirements, for example, only a minority of participants supported requiring 20 or more weekly hours or terminating people’s Medicaid benefits for noncompliance. Mandatory premium policies were unpopular. We also found that most people in a state that pursued Medicaid work requirements and premiums would not have supported the policy features that were enacted. Policymakers should take care to understand public preferences for both general policies and specific elements, and they should seek the views of program enrollees who will experience the effects of changes to benefits.

## Notes

Supplement.
Data Sharing Statement
