# Supplementary material for: Public Views on Medicaid Work Requirements and Mandatory Premiums in Kentucky
Source: JAMA Health Forum. 2023 Oct 20;4(10):e233656. doi: 10.1001/jamahealthforum.2023.3656 (PMC10589806; doi:10.1001/jamahealthforum.2023.3656)
Supplement: Supplement. — Data Sharing Statement [file jamahealthforum-e233656-s001.pdf]

# Data Sharing Statement

Underhill. Public Views on Medicaid Work Requirements and Mandatory Premiums in Kentucky. *JAMA Health Forum*. Published October 20, 2023.
doi:10.1001/jamahealthforum.2023.3656

**Data**
**Data available:** Yes
**Data types:** Deidentified participant data
**How to access data:** kunderhill@cornell.edu
**When available:** With publication

**Supporting Documents**
**Document types:** None

**Additional Information**
**Who can access the data:** Researchers whose proposed use of the data has been approved
**Types of analyses:** For approved research purposes
**Mechanisms of data availability:** After approval of a proposal
